# Age-Related Hearing Loss and the Factors Determining Continued Usage of Hearing Aids among Elderly Community-Dwelling Residents

**DOI:** 10.1371/journal.pone.0073622

**Published:** 2013-09-23

**Authors:** Kunio Mizutari, Takehiro Michikawa, Hideyuki Saito, Yasuhide Okamoto, Chieko Enomoto, Toru Takebayashi, Kaoru Ogawa, Yuji Nishiwaki

**Affiliations:** 1 Department of Otolaryngology-Head and Neck Surgery, Keio University School of Medicine, Tokyo, Japan; 2 Division of Otolaryngology, National Center for Child Health and Development, Tokyo, Japan; 3 Department of Preventive Medicine and Public Health, Keio University School of Medicine, Tokyo, Japan; 4 Environmental Epidemiology Section, Center for Environmental Health Sciences, National Institute for Environmental Studies, Tsukuba, Japan; 5 Department of Environmental and Occupational Health, Toho University School of Medicine, Tokyo, Japan; University of Salamanca- Institute for Neuroscience of Castille and Leon and Medical School, Spain

## Abstract

While hearing aids are recommended for people with age-related hearing loss, many with impaired hearing do not use them. In this study, we investigated how many elderly people in the study area needed hearing aids, and the factors that determined continued wearing of the devices. The study area was Kurabuchi Town, Japan, where 1,437 residents (those aged 65 years or over) were eligible for participation in the study; 1,414 participated, of whom, 103 (7.3%) were already using hearing aids at the start of the study. After the primary screening, hearing aids were lent to 68 participants (4.8%) who did not already have one, 38 of whom (60.3% of the borrowers, representing 2.7% of the total aged population) went on to wear the hearing aid continuously. The Hearing Handicap Inventory for the Elderly (HHIE) score was significantly elevated among these 38 participants. This study indicated that hearing aids are of potential benefit to many local residents. Multivariate logistic regression revealed that HHIE scores were associated with the extent of HA usage. The adjusted odds ratio for a 1-unit increase in HHIE score was 1.08 (95% confidence interval: 1.02–1.14). Programs like this, in which people with impaired hearing are identified at the local level and given appropriate assistance, are useful models for future use in societies with aging populations.

## Introduction

Age-related hearing loss (ARHL) is one of the most common disabilities in aged populations, and its prevalence is increasing: in the United States, 35% of individuals between 65 and 79 years old report hearing impairment; for those aged 80 and older, the corresponding figure is 53% [1]. According to the World Health Organization (WHO), ARHL is the top cause of moderate disability in people over the age of 60, with hearing aids (HAs) being the recommended treatment method [2]. Many reports describe the psychological effects of wearing a HA in addition to the auditory and acoustic effects [3], [4]. However, the proportion of hearing-impaired people who wear HAs even in developed countries is still low, and reports indicate that more than half of the hearing impaired do not wear a HA [5], [6], [7]. This is because many people find HAs too expensive, are embarrassed to wear them, or have not received proper information or guidance about wearing one [8], [9].

We conducted a field study in 2007–2008 dubbed Program of Education and Aid for the Community-dwelling Elderly (PEACE study), in which we investigated a broad range of health parameters, including quality of life and depression status; we also instituted an education program for the community-dwelling elderly residents of the area where the study was carried out. As part of PEACE study, we screened community-dwelling elderly people for age-related hearing loss in the community rather than at medical institutions such as hospitals. The expense of purchasing and maintaining a HA is a burden in all countries, including developed countries, and this greatly influences the rate of HA usage [8]. In this study, by providing HAs free of charge to local residents with hearing impairment above a certain level, we eliminated the bias brought about by the burden of expense. Firstly, we investigated the basic questions of how many community-dwelling elderly adults could potentially benefit from the provision of HAs, and how many would actually use them. Secondly, we examined the factors that determined the continued wearing of HAs.

## Materials and Methods

The PEACE study was carried out in 2007–2008 in Kurabuchi Town, Takasaki City, Gunma Prefecture (approximately 100 km north of Tokyo), Japan. Four of the town's 8 wards were covered in 2007 and the remaining 4 wards in 2008. Kurabuchi is in a rural, mountainous area, where a quarter of the population work in the primary sector of industry [10]. The population of the town is approximately 4,800, with residents aged 65 years or older accounting for about 30% of the total. The study was conducted with the cooperation of the town authorities on the basis of an agreement drawn up in advance.

Before the program commenced, public health nurses and local welfare commissioners visited the homes of all registered residents aged 65 years or older and identified 1,437 of them as eligible for participation in this study, after excluding those who were deceased, hospitalized, institutionalized, or who had moved out of the area at the time of the visit. Twenty-three of those eligible for participation declined to participate after their written informed consents were provided, so the total number of participating subjects was 1,414. Baseline health information was collected from all subjects in face-to face interviews. Information was also collected on demographic variables, lifestyle variables (education, living situation, smoking and drinking habits), self-rated general health, and HA ownership. Subjects were also asked to respond to the Hearing Handicap Inventory for the Elderly (HHIE). The HHIE is a self-assessment tool for evaluating the emotional and social problems elderly people experience because of hearing loss. It consists of 25 questions, is widely used and is well validated [11]. Each question has 3 response options (no [score: 0], sometimes [score: 2], and yes [score: 4]). The total score ranges from 0 to 100, and the higher the score, the greater the perceived hearing handicap. HHIE screening version (HHIE-S) consists of 10 questions (the total score ranges from 0 to 40), is also widely used. To minimize interviewer bias, training sessions were given to the interviewers before the interview survey commenced, and structured questionnaires were used in the interviews. Printed question cards were prepared for subjects with impaired hearing, and the interviewers read out the questions for those with impaired vision.

The program consisted of primary screening for ARHL, detailed assessment of hearing level, and the fitting and provision of HAs. The primary screening was carried out via the above-mentioned interview survey and objective medical examinations. The medical examinations, which included use of the Revised Hasegawa Dementia Scale (HDS-R), were performed in each of the 8 wards' community centers over a period of 11 days. The subjects were defined as positive for ARHL if they met any of the following criteria: 1) inability to hear both a 30-dB hearing level (HL) signal at 1,000 Hz and a 40-dB HL signal at 4,000 Hz in at least 1 ear when tested with pure-tone air-conduction audiometry (the 2 signals used in this test are designated by the Japanese Industrial Safety and Health Law for workers' health examinations); 2) failure of both ears in the finger friction test; 3) selection of “A little difficulty” or “A lot of difficulty” in response to “Do you have difficulty hearing and understanding what a person says to you in a quiet room when they speak normally (even when you are wearing your HA)?” [12]; 4) possession of a HA; 5) a score of 8 or more on the HHIE-S. Items 1) and 2) were assessed in the medical examinations, and items 3) to 5) were included in the interview survey. Naturally, there was considerable overlap among the subjects defined as positive for ARHL. The HDS-R, which was included in the medical examinations, is a neurocognitive functioning test consisting of 9 simple questions. The total score ranges from 0 to 30, with higher scores indicating better neurocognitive function [13]. The HDS-R is the most widely used test for dementia screening of the elderly in Japan. Those classified as positive for ARHL in the primary screening were invited to detailed hearing assessments carried out by 2 otolaryngologists (KM and HS) specializing in hearing. These assessments included examinations of the external ear canal and eardrum, pure-tone audiometry, and speech audiometry, and were carried out over a period of 4 days in the town office. Pure-tone air-conduction audiometry was conducted in a separate quiet room by trained technicians with an audiometer (AA-79S, -74, RION Inc., Tokyo, Japan); subjects who used HAs were asked to remove them for the tests. To reduce the influence of ambient noise (24- to 29-dB sound pressure level [A]), circumaural earphones were used. The audiometer was calibrated regularly to comply with Japanese standards (JIS T1201-1:2000 type 3). Air conduction thresholds were measured in both ears at 125, 250, 500, 1000, 2000, 4000 and 8000 Hz. Speech discrimination tests were carried out with recorded test materials from the 67-S monosyllable list developed by the Japan Audiological Society. The test materials were first presented at 10 dB above the threshold level at 1000 Hz, and the sound pressure was gradually increased until the maximum speech recognition level was obtained. The otolaryngologists assessed subjects as eligible for HA provision if they met one of the following criteria: 1) pure-tone average (PTA) obtained by the formula 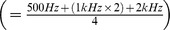 was 40 dB HL or worse in the better ear; 2) the subject expressed a strong desire for a HA, even if the average hearing level was slightly better than 40 dB HL. Subjects with an average hearing level of 70 dB or above were deemed ineligible for HA provision (the HAs on offer to the study participants were unsuitable for people with this level of hearing). In principle, HAs were fitted in the better ear, but when the hearing level was the same in both ears, they were fitted in the one with the higher maximum speech recognition score. This was done in an attempt to maximize the benefits of HA use; this measure distinguishes our study from others. Of the subjects eligible for HAs, only those who wanted one were provided with one (ear hook type, HB-DR5, RION Inc., Tokyo, Japan). One reason why we selected the HB-DR5 was that its maximum output sound pressure level is 126 dBSPL, which is sufficient to meet the needs of all but those with severe hearing loss. Another reason was that it allows for open fitting, which made it a convenient choice for the subjects with sloping hearing loss. Its data-logging feature was also an important consideration, because it allowed us to check the extent of actual usage of the HAs provided. Sound tests were performed with an LH-31 HA sound tester system (Rion Inc., Tokyo, Japan). NOAHlink (Himsa, Copenhagen, Denmark) was used to connect the fitting computer with the HAs, and Rionet Selector Software (Rion Inc., Tokyo, Japan) was used to adjust the devices. The prescribed target gain and maximum output level were determined on the basis of the NAL-NL2 formula installed in the fitting software. HA fitting was done by otolaryngologists and HA specialists (speech-language-hearing therapists and authorized HA technicians). At the initial HA fitting, 70–80% of the prescribed gain was used. The compression ratio was adjusted to within 1.5 to 2.0 to avoid word distortion. After the initial fitting, each participant tried the HA out for about 1 hour. Whenever this trial led to any complaints, we adjusted the sound accordingly. Once the participant was satisfied with the sound, the HA was provided free of charge after written consent had been obtained. The participants and their families were given counseling on the usage of the HAs, the mechanism of hearing loss, and the limitations of HAs. We traveled to the community one month after initial HA provision to conduct medical checks and HA fittings at the examination venue. Actual usage of each HA was checked with the data-logging feature. When the participant was found to have worn the HA continuously, the gain was increased near to the target gain calculated according to the NAL-NL2 formula. When the participant had not worn the HA continuously, the gain and maximum output level were adjusted to respond to the participant's complaints. At the same time, we explained the merits of HA usage to the participant and his/her family. If the participant was still reluctant to wear the HA, we provided further counseling both to the participant and his/her family. In cases where our best efforts to convince the participant and family of the benefits of wearing a HA failed, we accepted the device back. One month after the second fitting, we traveled to the community again and repeated the procedures. We made subsequent follow-up visits at least once every 6 months. Public health nurses and local welfare commissioners also visited the homes of the participants provided with HAs, and when they identified problems, they contacted us or the HA provider.

The study protocol was approved by the Ethics Committee of the School of Medicine, Keio University (Tokyo, Japan). Written informed consent have been obtained from all participant and all investigation have been conducted according to the principles expressed in the Declaration of Helsinki.

### Statistical analysis

The proportions of HA ownership before and after the program were calculated for the whole group and according to age category. The participants provided with HAs were divided into 2 groups for further analysis: those who used their HAs for an average of less than 1 hour per day, and those who used them for more than 1 hour per day, as determined by the devices' built-in data logs (the former group included those who returned the provided HAs within 6 months). We used the chi square test to analyze sex, educational background (high school or higher/up to junior high school), and living situation (living with family/living alone), and the Student's t-test analyze age. We also performed a non-parametric test (the Mann-Whitney test) on the HHIE scores, HDS-R scores, average hearing levels, and best speech discrimination levels of the participants provided with HAs. Finally, we used multivariate logistic regression to investigate the influence of various factors. In this analysis, HA usage of more than 1 hour per day was counted as an objective variable; the explanatory variables were: age category (65–69/70–79/80-), sex, educational category, living situation, HHIE scores (continuous), HDS-R scores (continuous), average hearing level (continuous), and best speech discrimination level (continuous). Stata version 11 (Stata Corp., College Station, TX, USA) was used for all data analyses.

## Results

**Figure 1:**
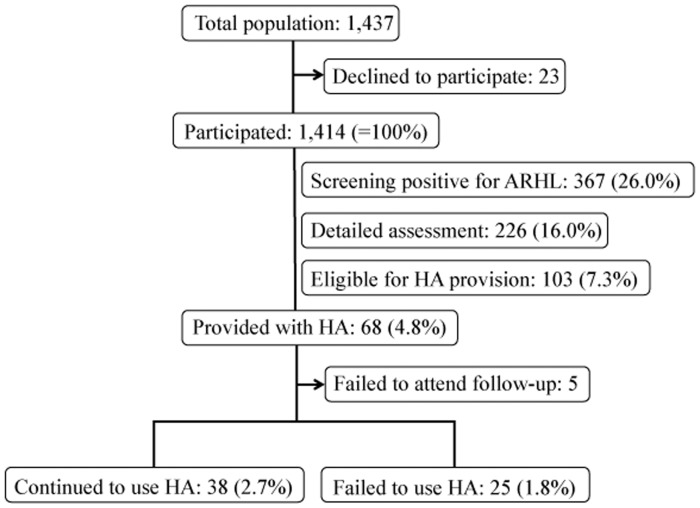
Procedure for the study along with the selection process, dropouts, and exclusions. ARHL  =  age-related hearing loss, HA  =  hearing aid.

Of the 1,414 participants, 639 (45.2%) were men, and the subjects aged 80 years or older constituted 32.0% of the total study population (Table 1). A total of 367 subjects (26.0%) were found to be positive for ARHL in the primary screening on the basis of the criteria listed under Materials and Methods: 158 subjects met criterion 1), 137 criterion 2), 142 criterion 3), 103 criterion 4), and 143 criterion 5) (as is clear from the figures, many of the subjects met more than one of the criteria). Of these 367 subjects, 226 (16.0% of the total number of subjects) participated in the detailed assessment of hearing level; of these 103 (7.3%) were found to be eligible for HA provision, and 68 (4.8%) were provided with HAs (Fig 1). The main reason why only around 60% of the subjects found to be positive for ARHL in the primary screening (226 out of 367) participated the detailed assessment was that the detailed assessments were carried out over a period of only 4 days, while the primary screening had been carried out over a period of 11 days. Of the 68subjects provided with HAs, 67 subjects had PTA of 40 dB HL or worse, and only 1 with PTA of 35 dB expressed a strong desire for a HA. One subject was excluded from HA provision because of PTA of above 70 dB. Ten subjects (0.7%) already owned HAs but had not been using them (the devices had not been fitted by specialists); for the other 58 (4.1%), HA use was a new experience. Before the program started, a total of 103 subjects (7.3%) already owned HAs, so as a result of the program the proportion of subjects who owned HAs increased from 7.3% to 11.4% (Table 2). For those subjects aged 75 years or older, the corresponding increase was from 11.8% to 17.7%.

**Table 1:** Characteristics of the study population (n = 1,414).

		Number (%)
Sex	Men	639 (45.2)
	Women	775(54.8)
Age (years)	65–69	299 (21.2)
	70–74	341 (24.1)
	75–79	321 (22.7)
	80-	453 (32.0)
Living alone		163 (11.8)*
Education	high school or higher	323 (23.9)*
Current smokers		171 (12.6)*
Current drinkers		397 (29.3)*
Self-rated poor health		159 (11.3)*

* data on some of the participants were unavailable.

**Table 2:** Ownership of hearing aids (whole group and by age category).

		Number (%)
**Whole group**	Before program	103 (7.3)
(n = 1,414)	After program	161 (11.4)
	Difference	58 (4.1)
**75 years or older**	Before program	91 (11.8)
(n = 774)	After program	137 (17.7)
	Difference	46 (5.9)
**below 75 years**	Before program	12 (1.9)
(n = 640)	After program	24 (3.8)
	Difference	12 (1.9)

Of the 68 subjects provided with HAs, only 63 were used in later analyses, because 5 did not attend the follow-up examinations. Of these 63 subjects, 16 (including 1 who had died) returned the HA because they did not want to use it. Eight of these said they felt there was no benefit in using a HA, 2 felt the HA was too loud, and 5 gave other reasons: 1 said the HA caused itchiness, 1 that it was impossible to insert the HA without help, 1 that it was inconvenient to wear the HA with glasses, 1 that the device caused hair problems, and the other simply did not want to use this kind of device. After 6 months, 9 of the remaining subjects had used the provided HAs for less than 1 hour per day as recorded by the HA data log, whereas 38 subjects (60.3% of the borrowers, 2.7% of the total aged population) had used theirs for an average of more than 1 hour per day.

The baseline characteristics of the study participants who borrowed HAs are shown in Table 3 in relation to their HA usage. With regard to whether or not the HA was used continuously, no significant differences were observed in terms of age, sex, educational background, living situation, cognitive function, or best speech discrimination level. On the other hand, in terms of hearing levels among participants who borrowed HAs at the time of the primary screening, with p = 0.08 considered statistically significant, the hearing threshold tended to be higher among those who used their HAs continuously. Furthermore, both the social and emotional HHIE scores were significantly higher in this group than among those who used their HAs for less than 1 hour or who returned them within 6 months.

**Table 3:** Baseline characteristics of study participants according to hearing-aid usage.

		Able to use HA	Unable to use HA	*p*-value
		(n = 38)	(n = 25)	
Age (years)	65–69 (SD)	3 (7.9)	1 (4.0)	
	70–79 (SD)	15 (39.5)	10 (40.0)	
	80- (SD)	20 (52.6)	14 (56.0)	0.82*
	average (SD)	80.3 (6.6)	79.8 (6.0)	0.78**
Sex	female(%)	15 (39.5)	14 (56.0)	
	male(%)	23 (60.5)	11 (44.0)	0.20*
Education	high school or higher (%)	6 (15.8)	7 (28.0)	
	up to junior high school (compulsory education) (%)	32 (84.2)	18 (72.0)	0.24*
Living situation	with family member(s) (%)	33 (89.2)	22 (91.7)	
	alone (%)	4 (10.8)	2 (8.3)	0.75*
HHIE score (full version, 25 questions)	Median (interquartile range)	14 (4–38)	0 (0–10)	<0.01***
HHIE-social score (12 questions)	Median (interquartile range)	9 (4–24)	0 (0–8)	<0.01***
HHIE-emotional score (13 questions)	Median (interquartile range)	3 (0–14)	0 (0–4)	0.01***
HDS-R score	Median (interquartile range)	25 (23–28)	25 (22–28)	0.54***
Average hearing level (dB HL)	Median (interquartile range)	54.4 (45.0–61.3)	48.8 (43.8–53.8)	0.08***
Maximum speech descrimination score (%)	Median (interquartile range)	60 (45–65)	55 (45–70)	0.67***

* χ2 test, ** Student's t-test, *** non-parametric test (Mann-Whitney test).

HHIE  =  Hearing Handicap Inventry for the Elderly, HDS-R  =  the Revised Hasegawa Dementia Scale.

Multivariate logistic regression revealed that only HHIE scores were associated with the extent of HA usage (Table 4). The adjusted odds ratio (OR) for a 1-unit increase in HHIE score was 1.08 (95% confidence interval [CI]: 1.02–1.14). When HHIE social scores and HHIE emotional scores were used instead of HHIE total scores, the corresponding ORs (95% CI) were 1.13 (1.04–1.24) and 1.16 (1.02–1.31), respectively. The social and emotional scores were highly correlational (r = 0.86), so we did not include these two scores simultaneously in the model. Average hearing level (adjusted OR: 1.09, 95% CI: 0.99–1.20) and best speech discrimination level (1.03, 0.99–1.07) showed a marginally significant association.

**Table 4:** Factors influencing hearing-aid usage in 63 subjects.

		Adjusted Odds Ratio
Age (years)	65–69	1
	70–79	0.84 (0.03–22.82)
	80-	0.63 (0.02–15.83)
Sex	female	1
	male	3.05 (0.78–11.95)
Education	high school or higher	1
	up to junior high school (compulsory education)	3.37 (0.66–17.26)
Living situation	with family member(s)	1
	alone	1.21 (0.14–10.05)
HHIE (full version, 25 questions)		1.08 (1.02–1.14)
HDS-R		1.10 (0.93–1.30)
Average hearing level		1.09 (0.99–1.20)
Maximum speech discrimination score		1.03 (0.99–1.07)

All factors in the table were included in the model.

HHIE  =  Hearing Handicap Inventry for the Elderly, HDS-R  =  the Revised Hasegawa Dementia Scale.

## Discussion

Before the program, 7.3% of our study population owned HAs, with 11.8% of those aged 75 years or above owning one. These ownership proportions are lower than those reported in Western countries [12], [14], but they are close to the proportions reported in other Japanese populations [15]. Whereas HA provision is covered by public health insurance in some European countries, e.g. the UK, France, Denmark, and the Netherlands, HAs themselves are not covered by the Japanese health insurance system. This may account for the relatively low proportion of ownership in Japan.

Very few studies have investigated the extent to which HA ownership by community-dwelling residents is increased by intervention [16]. Davis et al. invited men and women registered with a general practice in Cardiff, UK to audiometric tests, and those whose hearing level was greater than or equal to 30 dB in the worse ear were offered HAs [5]. As a result HA use increased from about 3% to over 9%. Stephens et al. carried out an intervention study using screening questionnaires and audiometric tests at two practices in West Glamorgan, UK, as a result of which the rate of HA use increased from 7% to 24% in one practice and from 8% to 22% in the other; again, the cutoff point for provision of a HA was a hearing level greater than or equal to 30 dB in the worse ear [17]. The study populations in the above 2 studies were residents aged between 50 and 65; adults older than 65 were not included. Wilson et al., on the other hand, carried out interviews and audiometric tests on 322 patients aged 65 and over who responded to an invitation to attend [18]. Of the 322 patients, 34 already had HAs. HAs were recommended for a further 142 patients who had a hearing level in the better ear of 35 dB or more, and 69 of these (48.9%) accepted the recommendation. HA use thus increased from 11% to 32%. In our study, ownership rose from 7.3% to 11.4%, and from 11.8% to 17.7% among those aged 75 or over. We used a hearing level of 40 dB or more in the better ear as the eligibility criterion for HA provision, which was higher than the cutoff levels used in other studies. We could probably have found a larger number of subjects eligible for HA provision if we had included residents aged under 65 in the study population, but the Japanese aversion to signs of age led us to believe that younger subjects would not accept HAs. This aversion can be seen in other countries as well, of course: Gussekloo et al. screened 454 German subjects aged 85 or over and provided HAs to those who needed them [19]; even among such an elderly population, only 56 (12.3%) accepted the devices for first-time use. Cost may also have a bearing on whether people will accept HAs or not. In our program, we provided HAs free. Had we charged for them, the acceptance rate would probably have been lower.

Our results also show that hearing-impaired people with high HHIE scores used HAs continuously. HHIE scores have been reported in a previous study to be a reliable factor for predicting the wearing of HAs [20]. However, that study was based on patients examined at clinics with a chief complaint of hearing loss, and we can assume that the patients were thus highly motivated to wear HAs. In contrast, the analyses in this study were based on data collected on elderly people living in a community that we visited, and as a result, we believe that our results more closely reflect the actual situation of elderly people. Moreover, since we used the data-logging feature built into the HAs to collect our data, we surmise that our data are more accurate than those collected via a questionnaire. Although our study has the strengths listed above, the study design also has limitations: one is that the audiological examinations we conducted were performed at community centers in the study area, so we could not perform detailed HA sound tests such as real-ear probe-microphone verification of HA gain and maximum output. Although real-ear measurements of HA sounds are widely used in clinics, we were unable to do them in this study for logistical reasons. Therefore, it was difficult for us to evaluate the true HA sounds in the real ear. Of the 16 subjects who returned HAs, 10 did so for reasons associated with HA sounds: 8 said they felt there was no benefit in using a HA, and 2 felt the HA was too loud. If we had been able to use a real-ear measurement system in this study, some of them might use have continued using their HAs. Such detailed tests should be carried out in future studies. Another limitation is the relatively long periods between follow-up visits. We were able to visit the research area only once a month, even after the initial fitting process. We usually see patients every week or every two weeks immediately after they are fitted with HAs at hospital to adjust HA gain and maximum output. Again, if we had been able to make more frequent follow-up visits, the number of participants who returned HAs might have been smaller.

Using HAs correctly is known to lead to decreased HHIE scores [21], [22]. In an earlier study, we reported that high HHIE scores are a risk factor in the development of depression in elderly people [23], so we believe that HHIE screening of community-dwelling elderly people is useful.

In this study, 38 of the subjects who borrowed a HA used it every day. This figure represents only 2.7% of the total elderly population in the study area, which is a very small figure when you consider that 7.3% of the study population already owned a HA. However, by actively getting involved on a community level, rather than a hospital level, we were able to help people with hearing difficulties. HHIE screening can be conducted easily and does not require any special skills or equipment. By visiting communities and introducing HHIE screening we can more effectively identify people with hearing impairment who are in need of detailed hearing assessments and otological examinations and can then recommend HAs for those who need them. We also believe that this kind of direct approach is cost-effective.

In this study, priority was given to using the limited research budget to lend HAs to as many participants as possible. Although we know that wearing HAs in both ears is preferable from the perspective of auditory localization in noisy environments [24] when ARHL is the same in both ears, we were able to provide only one HA per participant for use in the better ear. Even with only one HA, the rate of continued usage among the participants who were provided with one was high: around 60%. Our results showed that fitting a HA in one ear led to satisfactory outcomes, which is an important finding from the point of view of medical economics: although funding for HAs may be limited, they can be distributed to more people with hearing impairment. Public provision of even only one HA per person should lead to improvements in the quality of life (QOL) of many hearing-impaired people, because the use of HAs has been reported to improve hearing-related QOL [25].

## Conclusions

We were able to identify subjects in need of HAs and increase usage of the devices. Programs such as ours, in which the HHIE is used to screen elderly people for ARHL in their local communities so that appropriate assistance can be provided, should prove useful in all societies with aging populations.
